# Isolation and Characterization of Polymorphic Microsatellite Markers from the Chinese Medicinal Herb *Atractylodes macrocephala* (Asteraceae)

**DOI:** 10.3390/ijms131216046

**Published:** 2012-11-28

**Authors:** Li Zheng, Zhong-Da Shao, Zong-Chao Wang, Cheng-Xin Fu

**Affiliations:** 1 Key Laboratory of Conservation Biology for Endangered Wildlife of the Ministry of Education, College of Life Sciences, Zhejiang University, Hangzhou 310058, China; E-Mails: zhengli0420@163.com (L.Z.); szd27112@yahoo.com.cn (Z.-D.S.); yqszd620207@zju.edu.cn (Z.-C.W.); 2 Laboratory of Systematic & Evolutionary Botany and Biodiversity, Institute of Plant Sciences, and Conservation Center for Gene Resources of Endangered Wildlife, Zhejiang University, Hangzhou 310058, China

**Keywords:** *Atractylodes macrocephala*, microsatellites, genetic diversity, Chinese medicinal herb

## Abstract

*Atractylodes macrocephala* Koidz. (Asteraceae) is an economically important Chinese medicinal herb. In this study, 15 polymorphic microsatellite markers were developed from *A. macrocephala* using the compound microsatellite marker technique. Levels of polymorphism within the 15 markers were assessed using 83 individuals from two wild and two cultivated populations in China. The number of alleles per locus ranged from 2 to 20, with an average of 9.9 alleles. Observed and expected heterozygosities ranged from 0.083 to 1.000 and from 0.097 to 0.938, respectively. These markers will be valuable for germplasm classification and identification, as well as for assessing the genetic diversity and spatial genetic structure among wild and cultivated populations of *A. macrocephala*.

## 1. Introduction

*Atractylodes macrocephala* Koidz. (Asteraceae) is a perennial herb that is endemic to several provinces in China. The dried rhizome of *A. macrocephala*, also known as Atractylodes Macrocephala Rhizoma (common name: “Baizhu” in Chinese and “Byakujutsu” in Japenese), is widely used in traditional herbal remedies in Asia [1,2]. It was reported as a nutrient for energy and for the treatment of dyspepsia and anorexia [3]. The essential oil of Atractylodes Macrocephala Rhizoma possesses an array of biologically active secondary compounds, including those that are gastroprotective [4], anti-inflammatory [5], anti-carcinogenic [6] and anti-microbial [7] activity according to pharmacological investigations. Due to over-exploitation and habitat destruction, *A. macrocephala* is now rare and threatened in the wild, and has even disappeared in many locations, e.g., Zhejiang Province [8]. Three populations QM (Qimen County, Anhui Province) [9], JL (Jiulong Mountain, Hunan Province) and MC (Micang Mountain, Shaanxi Province) are the largest known extant wild populations of the species, and they are sporadic in these regions (according to our investigation). Cultivation of the species began in Zhejiang Province during the 16th century, and has since been introduced into fifteen other provinces in China [3]. Eastern Zhejiang Province is well known for its high quality Atractylodes Macrocephala Rhizoma, while Bozhou City in Anhui Province is now the largest producer.

In the field, *A. macrocephala* is a perennial herb, which can reproduce sexually through cross-pollination [9]. It is cultivated as a biennial crop by seed in China and is generally harvested after the fruits ripen. However, the germplasm resources of cultivated populations of the species have degraded over time as a consequence of domestication processes. Root rot and sclerotium blight have become the main causes of yield reduction of this medicinal crop in many areas [3]. It was reported that the erosion of the genetic variability might result in reduction of the plasticity of crops to respond to changes in climate, pathogen populations, agricultural practices, or quality requirements [10]. Therefore, assessment of genetic diversity by molecular markers is important not only for crop improvement efforts, but also for efficient management and conservation of plant genetic resources. However, very little is currently known about genetic variation in wild or cultivated *A. macrocephala* populations [11]. Here, we report the isolation and characterization of the first set of polymorphic microsatellite markers for this species and expect these to provide a useful tool for population genetic studies.

## 2. Results and Discussion

A total of 91 primer pairs were designed from microsatellite sequences isolated from the microsatellite-enriched libraries. Twenty out of them successfully yielded one clear band, while the others produced multiple bands or no amplification (detected by 2.0% agarose gel electrophoresis). These primers were then chosen to test for polymorphism.

Eighty-three individuals of *A. macrocephala* (Supplementary Table S1) from two cultivated populations (Panan County, Zhejiang Province and Pingjiang County, Hunan Province) and two wild populations (Jiulong Mountain, Hunan Province and Micang Mountain, Shaanxi Province) in China were employed to test the efficiency of the locus-specific primer pairs and to characterize these microsatellite markers. Among the 20 primer pairs, 15 were polymorphic and 5 were monomorphic within all four populations of *A. macrocephala*, though these monomorphic markers could be potentially useful for other populations not tested [12] or other related species. The allele sizes of the 20 microsatellite loci ranged from 77 to 297 bp, and the amplification products were within the expected size range (Table 1).

As expected, amplification using these 15 primer pairs resulted in one or two alleles per individual of this diploid species, suggesting that each primer pair amplified its locus-specific microsatellite region. The number of alleles per locus (*N*_a_) ranged from 2 to 20 (mean: 9.9) among the four populations (Table 1). The observed (*H*_o_) and expected heterozygosities (*H*_e_) ranged from 0.083 to 1.000 (mean: 0.549) and from 0.097 to 0.938 (mean: 0.657), respectively (Table 2).

The mean number of the alleles per locus was 6.9 (range: 2–13), 6.5 (range: 2–12), 6.5 (range: 2–13) and 6.7 (range: 2–13) for population PA, PJ, JL and MC, respectively (Table 2). On average, the observed heterozygosities (*H*_o_) were 0.553 (range: 0.083–0.917), 0.603 (range: 0.167–0.958), 0.497 (range: 0.100–1.000) and 0.551 (range: 0.200–0.867), respectively (Table 2). The expected heterozygosities (*H*_e_) were 0.656 (range: 0.194–0.910), 0.658 (range: 0.223–0.885), 0.655 (range: 0.097–0.923) and 0.660 (range: 0.191–0.938), respectively (Table 2). For the two cultivated populations (population PA and PJ) and two wild populations (population JL and MC), the mean number of the alleles per locus was 6.7 (range: 2–13) and 6.6 (range: 2–13), respectively (Table 2). On average, the observed heterozygosities (*H*_o_) were 0.578 (range: 0.083–0.958) and 0.524 (range: 0.100–1.000), respectively (Table 2). The expected heterozygosities (*H*_e_) were 0.657 (range: 0.194–0.910) and 0.658 (range: 0.097–0.938), respectively (Table 2). The results showed that the degree of polymorphism among the four populations and between the cultivated and wild populations were not significantly different. The average number of alleles per locus and the mean expected heterozygosities for the four populations were similar. However, the mean observed heterozygosities of the two cultivated populations were higher than those of the two wild populations.

Nearly half of the fifteen analyzed loci showed significant deviation from the Hardy-Weinberg equilibrium (*p* < 0.05) in three populations (PA, PJ and JL), while just three loci significantly deviated from the HWE expectations in the wild population MC (Table 2). The results of null allele test using MICRO-CHECKER (*p* < 0.001) were similar to the test for Hardy-Weinberg equilibrium. Specifically, null alleles may be present at six loci in population PA (Am4, Am5, Am7, Am11, Am13 and Am15), four loci in population PJ (Am5, Am7, Am11 and Am15), four loci in population JL (Am7, Am11, Am14 and Am15) and two loci in population MC (Am12 and Am15). The departures from Hardy-Weinberg equilibrium, most of which were caused by heterozygote deficiency, are likely to be due to the presence of null alleles (Am7, Am11 and Am15). Alternatively, inbreeding in the cultivated populations and genetic drift among small, fragmented wild populations may explain our observations. Two pairs of loci in population PA (Am4 & Am7, Am2 & Am4) and two pairs in population PJ (Am2 & Am4, Am9 & Am10) were found to have significant LD (*p <* 0.01), while no significant LD was observed in any loci pairs in the two wild populations. Thus, LD is likely due to different population structure rather than to physical linkage of the markers.

## 3. Experimental Section

### 3.1. Isolation of Microsatellite Markers

The compound microsatellite marker technique based on a dual-suppression-PCR method was used to develop microsatellite markers for *A. macrocephala* in this study [13]. Two individuals from population PA (Supplementary Table S1) were employed to construct the microsatelite-enriched libraries. Total genomic DNA was extracted from silica-gel-dried leaf material using the modified CTAB method by Doyle [14]. The microsatellite-enriched libraries were constructed following Zhai *et al*. [15] except that we used ca. 500 ng of total genomic DNA and the *Hae*III and *Ssp*I (Takara) blunt-end restriction enzymes for both individuals. Fragments flanked by a microsatellite at one end were amplified from both the *Hae*III and *Ssp*I DNA libraries, and then the fragments whose size were between 400 and 1000 bp were purified and ligated into PMD18-T vector (Takara), and subsequently transformed into DH5α competent cells (Takara) as described by Yuan *et al*. [16].

A total of 223 positive clones were obtained and sequenced on an ABI Prism 3730 automated DNA sequencer (Applied Biosystems). One hundred and ninety-four sequences were found to contain (AC)_6_(AG)*_n_* or (TC)_6_(AC)*_n_* compound SSR motifs, of which ninety-one possessed unique sequences with sufficient flanking regions for designing specific primers. A specific primer (IP1) was designed using Primer Premier Version 5.0 [17]. Because there might be hundreds of binding sites of the compound SSR primer in the genome, the specificity of the primer pairs mostly depended on IP1. Therefore, IP1 was designed using the following criteria: (1) primer size between 18 and 22 bp in length; (2) amplicon size between 80 and 300 bp; (3) GC content between 35% and 65%; (4) base runs no more than four, especially no more than three base runs for G or C; and (5) the most important, *T*m value of IP1 was lower than the compound SSR primer and within 2 °C, because primer with lower *T*m value normally determines the specificity of the primer pairs. Finally, the primer pair with highest score given by Primer Premier was chosen.

All PCR amplifications were performed in a 15 μL reaction mixture containing 50–75 ng of genomic DNA, 0.75 U of *Taq* polymerase (Takara), 1.5 μL of 10 × PCR buffer with MgCl_2_, 1.2 μL of dNTPs (2.5 mM each), 0.1 μL bovine serum albumin (BSA, Takara), 0.5 μL of each primer (10 μM). PCR amplification conditions were as follows: initial denaturation at 95 °C for 5 min; 35 cycles of 30 s at 95 °C, 30 s at the optimized annealing temperature (Table 1), 30 s of elongation at 72 °C; ending with a 5 min extension at 72 °C. Fragment analysis was performed on a MegaBACE 1000 autosequencer (GE Healthcare Biosciences), and alleles were scored using the GeneMaker Software version 1.97 (SoftGenetics) as described by Yuan *et al*. [16].

### 3.2. Data Analysis

The number of observed alleles per locus (*N*_a_), as well as observed (*H*_o_) and expected (*H*_e_) heterozygosities, were calculated by CERVUS version 3.0.3 [18]. Hardy-Weinberg equilibrium (HWE) and linkage disequilibrium (LD) between all pairs of polymorphic loci were analyzed using GENEPOP version 4.0.7 [19]. The presence of null alleles at each locus was tested using MICRO-CHECKER version 2.2.3 [20].

## 4. Conclusions

The 15 novel microsatellite markers that we have developed for *A. macrocephala* showed high polymorphism among and within four populations of *A. macrocephala.* Thus, these markers are suitable for population genetics studies of this medicinal plant. In our ongoing research, we are applying them to investigate genetic diversity and spatial genetic structure over both cultivated and wild populations of this important medicinal species. Furthermore, we expect that these markers will facilitate future studies on the origin and evolutionary history of cultivated populations, as well as on the efficient conservation and sustainable exploitation of the *A. macrocephala* germplasms.

## Supplementary Information



## Figures and Tables

**Table 1:** Characteristics of 20 compound microsatellite loci developed for *Atractylodes macrocephala*.

Locus	Repeat motif	Primer sequence (5′ to 3′)	*T*_a_(*°*C)	Size range (bp)	*N*_a_	GenBank Accesion no.
Am1	(AC)_6_(AG)_7_	F: (AC)_6_(AG)_5_ R: TTACCACGATGAGCAAAAC	54	77–89	5	JX242500
Am2	(AC)_6_(AG)_5_GG(AG)_4_	F: (AC)_6_(AG)_5_ R: CTATTGCCACCTTCTTGC	52	225–275	18	JX242501
Am3	(AC)_6_(AG)_6_	F: (AC)_6_(AG)_5_ R: TATCGGGTACATCAGAGCA	50	166–172	2	JX242502
Am4	(TC)_6_(AC)_8_AT(AC)_2_	F: (TC)_6_(AC)_5_ R: ATACGCAAAACTCGAACC	58	123–149	12	JX242503
Am5	(TC)_6_(AC)_8_AT(AC)_2_	F: (TC)_6_(AC)_5_ R: AAGGTGGAGCTAGGAAATC	50	159–173	5	JX242504
Am6	(TC)_6_(AC)_9_ATAC	F: (TC)_6_(AC)_5_ R: TGGAATCGAATCCCTAAA	52	90–106	7	JX242505
Am7	(TC)_6_(AC)_11_AT(AC)_12_	F: (TC)_6_(AC)_5_ R: TTGAACCCTGTTGACCATA	56	232–264	16	JX242506
Am8	(TC)_6_(AC)_5_AT(AC)_4_	F: (TC)_6_(AC)_5_ R: GGTAGTAGAACCCAAGCAA	54	163–189	5	JX242507
Am9	(TC)_6_(AC)_19_	F: (TC)_6_(AC)_5_ R: CAGAAATATCGGAACTCCTT	52	206–242	18	JX242508
Am10	(TC)_6_(AC)_7_	F: (TC)_6_(AC)_5_ R: GGCAGCCATTACAACTCA	56	83–89	6	JX242509
Am11	(TC)_6_(AC)_14_TC(AC)_2_	F: (TC)_6_(AC)_5_ R: TATCCTTACTCGGACATTACA	50	260–290	12	JX242510
Am12	(TC)_6_(AC)_10_	F: (TC)_6_(AC)_5_ R: TCGAAATTCTTACCGTCAA	54	251–297	20	JX242511
Am13	(TC)_6_(AC)_5_	F: (TC)_6_(AC)_5_ R: ACGTTATCGTCCGAAATG	52	154–160	4	JX242512
Am14	(TC)_6_(AC)_5_(AT)_3_	F: (TC)_6_(AC)_5_ R: GGTAGTGGCTATTGGGACT	52	197–199	2	JX242513
Am15	(TC)_6_(AC)_10_AT(AC)_5_	F: (TC)_6_(AC)_5_ R: CTATGTTAGAAGGCTGGTGTT	50	205–241	17	JX242514
Am16	(AC)_6_(AG)_5_	F: (AC)_6_(AG)_5_ R: CCGTATCATGGGAGGTAA	52	132	1	JX964787
Am17	(AC)_6_(AG)_5_	F: (AC)_6_(AG)_5_ R: TTACCTTTGAGTTCTTTACACC	54	85	1	JX964788
Am18	(AC)_6_(AG)_5_	F: (AC)_6_(AG)_5_ R: CTATACCCACCAAGTCACAA	50	194	1	JX964789
Am19	(TC)_6_(AC)_7_	F: (TC)_6_(AC)_5_ R: CCAAGTAGGGTCCAGATTC	56	176	1	JX964790
Am20	(TC)_6_(AC)_5_	F: (TC)_6_(AC)_5_ R: AAAGCGGAATATGGTTTC	56	165	1	JX964791

*Note*: F = forward primer; R = reverse primer; *T*_a_ = annealing temperature; *N*_a_ = number of alleles per locus.

**Table 2:** Results of initial primer screening in four populations of *Atractylodes macrocephala.*

Locus	Population PA( *N* = 24 )			Population PJ( *N* = 24 )			Population JL (*N* = 20 )			Population MC(*N* = 15 )		

	*N*_a_	*H*_o_	*H*_e_	*N*_a_	*H*_o_	*H*_e_	*N*_a_	*H*_o_	*H*_e_	*N*_a_	*H*_o_	*H*_e_
Am1	4	0.333	0.472 n.s.	4	0.375	0.332 n.s.	3	0.300	0.273 n.s.	3	0.533	0.577 n.s.
Am2	12	0.792	0.846 n.s.	11	0.875	0.841 **	13	1.000	0.923 n.s.	11	0.667	0.899 n.s.
Am3	2	0.750	0.511 *	2	0.792	0.511 *	2	0.350	0.481 n.s.	2	0.533	0.497 n.s.
Am4	8	0.417	0.703 ***	9	0.917	0.847 n.s.	7	0.700	0.817 n.s.	10	0.800	0.853 n.s.
Am5	4	0.292	0.502 *	5	0.167	0.428 ***	3	0.350	0.606 *	3	0.333	0.297 n.s.
Am6	2	0.542	0.403 n.s.	5	0.667	0.569 n.s.	6	0.700	0.728 n.s.	4	0.600	0.646 n.s.
Am7	12	0.625	0.878 ***	9	0.583	0.846 ***	10	0.550	0.869 ***	10	0.667	0.814 n.s.
Am8	5	0.625	0.738 *	5	0.375	0.590 **	3	0.300	0.456 *	5	0.333	0.361 n.s.
Am9	12	0.875	0.847 n.s.	10	0.958	0.818 n.s.	9	0.800	0.864 n.s.	12	0.867	0.910 n.s.
Am10	4	0.750	0.724 n.s.	4	0.917	0.766 n.s.	5	0.800	0.792 n.s.	5	0.600	0.706 n.s.
Am11	10	0.500	0.854 ***	7	0.542	0.811 **	8	0.300	0.864 ***	6	0.600	0.823 *
Am12	11	0.917	0.882 n.s.	12	0.792	0.869 n.s.	13	0.750	0.904 *	13	0.667	0.938 *
Am13	3	0.083	0.194 *	4	0.333	0.535 *	2	0.100	0.097 n.s.	3	0.200	0.191 n.s.
Am14	2	0.333	0.383 n.s.	2	0.250	0.223 n.s.	2	0.150	0.296 *	2	0.400	0.460 n.s.
Am15	13	0.458	0.910 ***	9	0.292	0.885 ***	11	0.300	0.859 ***	12	0.467	0.924 ***
Mean	6.9	0.553	0.656	6.5	0.603	0.658	6.5	0.497	0.655	6.7	0.551	0.660

*Note: N* = sample size for each population; *H*_o_ = observed heterozygosity; *H*_e_ = expected heterozygosity; *N*_a_ = number of alleles per locus; Population PA = Panan population; Population PJ = Pingjiang population; Population JL = Jiulong population; Population MC = Micang population. *, ** and ***, Significant departures from Hardy-Weinberg equilibrium at *p* < 0.05, *p* < 0.01, *p* < 0.001, respectively; n.s. = not significant.
