# Supplementary material for: Isolation and Characterization of Polymorphic Microsatellite Markers from the Chinese Medicinal Herb Atractylodes macrocephala (Asteraceae)
Source: Int J Mol Sci. 2012 Nov 28;13(12):16046–52. doi: 10.3390/ijms131216046 (PMC3546677; doi:10.3390/ijms131216046)
Supplement: Supplementary file 1 [file ijms-13-16046-s001.pdf]

Supplementary Information

Table S1. List of vouchers of *Atractylodes macrocephala* used in this study. Vouchers are deposited in the Herbarium of Zhejiang University (HZU).

Code	Location	Altitude (m)	Longitude	Latitude	Voucher number
PA	Panan County, Zhejiang Province	520	120°27'41.99"E	29°05'12.88"N	ZL100901
PJ	Pingjiang County, Hunan Province	480	113°59'17.99"E	28°38'12.13"N	ZL110708
JL	Jiulong Mountain, Hunan Province	1150	111°04'03.39"E	26°38'06.81"N	ZL110713
MC	Micang Mountain, Shaanxi Province	1380	106°43'28.80"E	32°46'00.92"N	ZL110821

© 2012 by the authors; licensee MDPI, Basel, Switzerland. This article is an open access article distributed under the terms and conditions of the Creative Commons Attribution license (http://creativecommons.org/licenses/by/3.0/).
